# A Rare Case of Gastrointestinal Stromal Tumor with a Liver Metastasis Infiltrating the Inferior Vena Cava and Extending to the Right Atrium with an Early Recurrence after Surgical Extraction

**DOI:** 10.1155/2019/2623403

**Published:** 2019-02-05

**Authors:** Dimitrios Siamkouris, Marc Schloesser, Amr Yousef, Elmar Offers

**Affiliations:** Department of Cardiology, Dreifaltigkeits Hospital Lippstadt, Germany, Academic Teaching Hospital of Westfaelische Wilhelms University Muenster, Germany

## Abstract

Gastrointestinal stromal tumors (GISTs) are mesenchymal tumors of the gastrointestinal tract. The major cause of GIST is the presence of an abnormal form of tyrosine protein kinase (KIT) protein also known as CD117, which causes uncontrollable growth of the gastrointestinal cells. Most studies report incidences between 10 and 15 cases of GISTs per million. Metastases to the liver and peritoneum are the most frequent. We report a case of advanced GIST with a liver metastasis infiltrating the inferior vena cava (IVC) and extending to the right atrium in the form of a large, floating, isolated intracardiac liver metastasis with diastolic prolapsing through the tricuspid valve. This is a very rare manifestation. One week after heart surgery and removal of a 5 × 6 cm tumor mass from the right atrium and the IVC, echocardiography depicted an early recurrence.

## 1. Case

**Figure 1:**
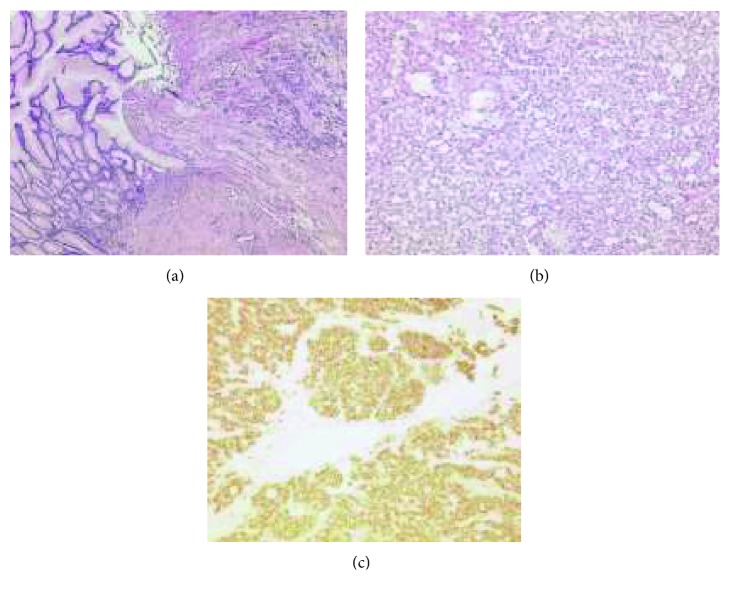
(a) Stomach ulcer with epithelioid GIST (50x, hematoxylin and eosin stain). (b) Gastric epithelioid GIST (100x, hematoxylin and eosin stain). (c) Membrane pattern of KIT immunostaining in epithelioid GIST (100x, CD117).

A 51-year-old female patient was admitted about 4 years ago to the emergency room for nausea, vomiting, dizziness, melena, and syncope. The patient's history indicated upper gastrointestinal bleeding, and immediate esophagogastroduodenoscopy (EGD) revealed an ulcerating tumor in the pyloric antrum with blood oozing, strongly suspected for a gastric GIST. Haemostasis was achieved after endoscopic injection of epinephrine and subsequent adequate blood transfusion due to haemorrhagic shock led to the stabilisation of the patient. The histological diagnosis was ulcerating epitheloid GIST (Figure 1). Immunohistochemically, tumor cells were strongly positive for CD117, platelet-derived growth factor receptor-alpha (PDGFRA), discovered on GIST-1 (DOG1), and Bcl-2. CD34 was not evident. The mitotic rate was 8/50 high-power fields (HPF), and the Ki67-index/proliferation rate was estimated at 5%. The molecular pathological examination showed duplication in exon 11 of the KIT gene. The abdominal computed tomography (CT) scan showed no lymph node, liver, or bone metastasis. The pT2 M0 R0 (TNM classification) staged tumor was operated successfully with an open 2/3 stomach resection with a Roux-en-Y anastomosis and jejunojejunostomy. The 3.5 cm tumor was completely excised with edges clear of infiltration and no tumor infiltration of the serosa. The postoperative course was very satisfactory with no sequelae, and no adjuvant imatinib therapy was administrated after multidisciplinary treatment planning. The patient could be discharged 3 weeks after admission with the recommendation for abdominal (CT) scan and EGD every 6 months for the next 5 years.

**Figure 2:**
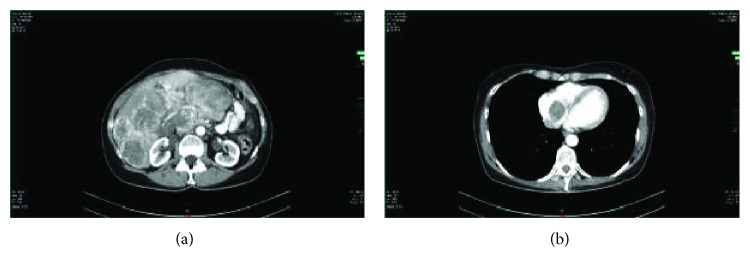
(a) Abdominal CT scan with IV contrast showing multiple liver metastases. (b) Chest CT scan showing a filling defect within the contrast-enhanced right atrium which was initially thought to be a large thrombus.

**Figure 3:**
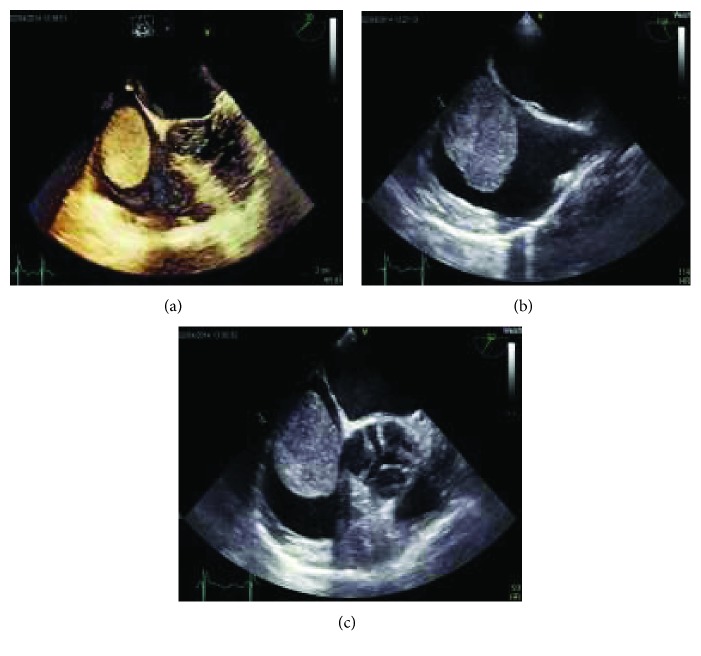
Large atrial mass in (a) the TEE 3D 4-chamber view, (b) the TEE 2D bicaval view, and (c) the TEE 2D short-axis view, conclusive for a mobile large atrial mass.

**Figure 4:**
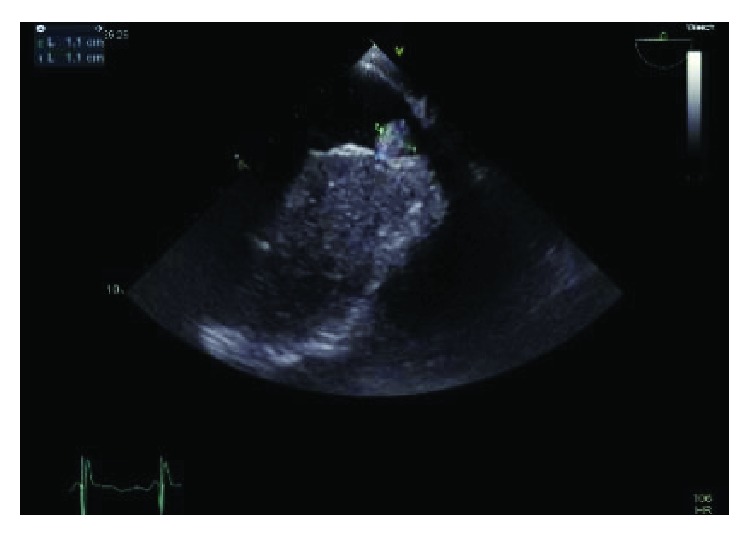
TEE reveals a similar mass in the IVC.

15 months later and in the scope of the follow-up examination, the patient complained for discomfort and slight pain in the right upper abdomen. The abdominal ultrasonography revealed multiple liver metastases, and the EGD confirmed a recurrence of GIST in the anastomosis. The abdominal and chest CT scan (Figure 2) confirmed diffuse liver metastases and revealed an encircling wall architecture of the GIST around the hepatic hilum with a partial obstruction of the common bile duct and a shifting of the portal vein without signs of portal vein thrombosis. However, no icterus was present. The CT scan revealed furthermore a suspected large thrombus in the IVC and right atrium. There was no evidence of lymph node, bone, or lung metastasis. The transthoracic (TTE) and subsequent transoesophageal echocardiography (TEE) disclosed the presence of a 5.3 × 3.4 cm large mass in the right atrium with diastolic prolapsing through the tricuspid valve, without any clear attachment to the atrial wall, with an inhomogeneous appearance, and without vacuolisation (Figure 3), along with a similar 1.1 cm large mass in the IVC (Figure 4) with a suspected but no clear continuation between these two masses even after free style image acquisition. The patient denied any angina or dyspnoea. Anticoagulant therapy with low molecular weight heparin showed no improvement within few days, ruling out a thrombus formation and suggesting intracardiac metastasis. Due to recurrent electrocardiogram (ECG) alternations in the precordial leads suggestive of intermittent lung embolism, the imminent right ventricular diastolic flow obstruction with a resulting obstructive form of a cardiogenic shock, and because of the young age of the patient and the potential good response to imatinib therapy, she was referred to a cardiothoracic clinic.

**Figure 5:**
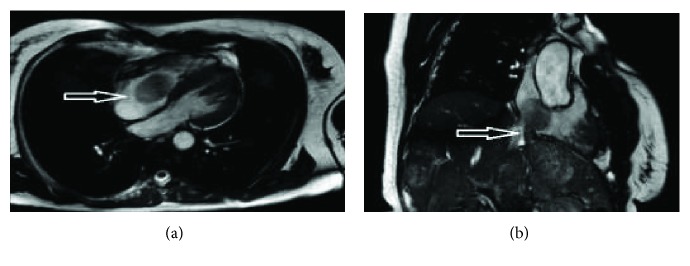
Cardiac MRI confirms the mass in (a) the right atrium with prolapsing through the tricuspid valve and (b) the contiguous extension of the liver metastasis to the IVC and right atrium (white arrows).

**Figure 6:**
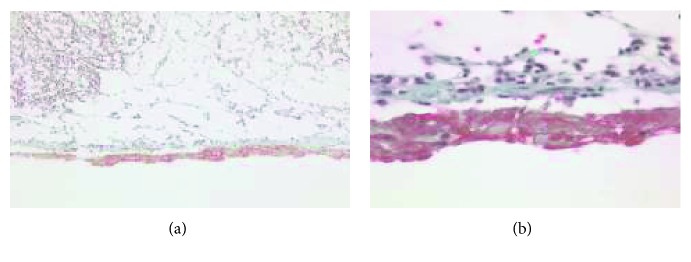
Goldner's trichrome histological staining method at 20x magnification (a) and 80x magnification (b) exhibiting the tumor in the upper layer, fibrosis in the middle green layer, and thrombosis in the lower red layer. Between the fibrotic band and the thrombosis, there are identifiable gut endothelial cells from the VCI.

**Figure 7:**
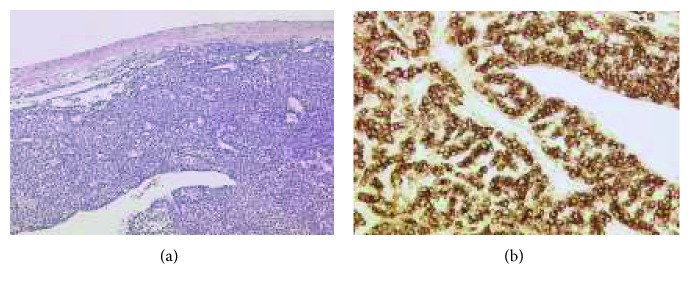
(a) Metastasis of epithelioid GIST in the right atrium of the heart (50x, hematoxylin and eosin stain). (b) Strong cytoplasmic pattern of KIT immunostaining in epithelioid GIST in the right atrium of the heart (200x, CD117 antibody).

**Figure 8:**
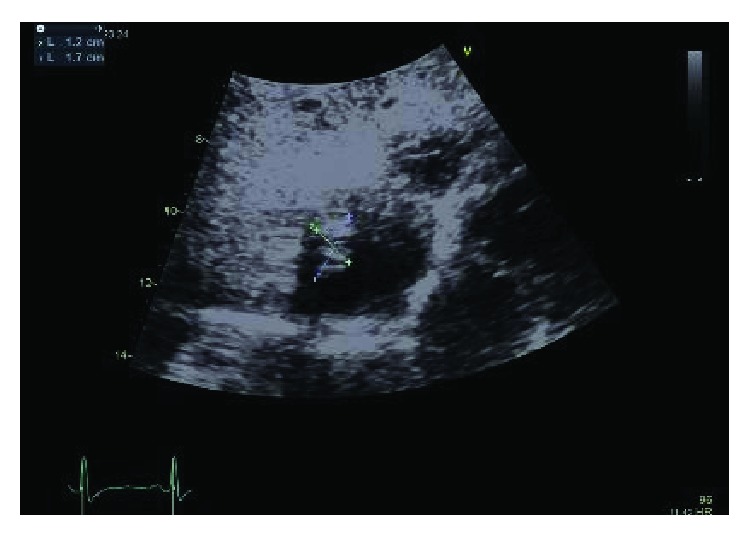
Tumor recurrence one week after surgical removal.

The patient underwent cardiac catheterization which revealed no obstructive coronary atherosclerotic plaque. Prior to surgery, cardiac magnetic resonance imaging (Figure 5) verified the cardiac findings of echocardiography but also clearly exhibited a propagation of a liver metastasis as a continuous mass to the IVC and right atrium with no adherence to the cardiac wall. Surgical resection of the tumor in the right atrium and IVC was performed using a minimally invasive anterior lateral access approach supplemented with video-assisted thoracoscopy. The 50 × 60 mm resected tumor with endothelial infiltration of the IVC, concomitant local thrombosis (Figure 6), and no endocardial and myocardial infiltration of the right atrium was histologically and immunohistologically identical to the prediagnosed GIST (Figure 7) and was a contiguous extension of a liver metastasis to the heart. The patient could recover from the operation and was treated with 400 mg imatinib daily. However, a week after surgery, transthoracic echocardiography (Figure 8) revealed a new mass in the right atrium indicative of early recurrence. The patient deteriorated over time due to a further progression of the GIST with icterus. The course was furthermore complicated by *Staphylococcus aureus* sepsis due to central venous catheter infection, and the patient finally died due to multiorgan failure.

## 2. Discussion

GISTs are rare, potentially aggressive forms of cancer that may develop anywhere in the GI tract [1]. Most studies report incidences between 10 and 15 cases of GISTs per million. Estimates suggest that the prevalence is over 10 times the incidence [2]. Surgical resection remains the aimed therapy of GIST. Complete resection of the tumor is the main predictor of the postoperative survival of the patient [1]. Despite successful surgical therapy, GISTs exhibit a high risk for metastatic relapse. The work of Hirota et al. and subsequently Heinrich et al. proved that most of the GISTs are caused by c-KIT somatic gain-of-function mutations followed by similar mutations in platelet-derived growth factor receptor-alpha (PDGFRA) [3, 4]. The initiation of KIT-selective tyrosine kinase inhibitor therapy such as imatinib and its successors improved the survival of these patients [5, 6]. However, the benefit in recurrence-free survival appears to be related to tumor size, with the most marked improvement in patients who had a large tumor (≥10 cm) [6]. In our case, the multidisciplinary tumor board conference involving pathologists, radiologists, surgeons, and medical oncologists as well as gastroenterologists, based on the positive surgical and clinical course of the patient and the so far scientific evidence at the time of treatment of our patient, which was not conclusive for adjuvant imatinib therapy in patients with locoregional GISTs and R0 surgery (i.e., an excision whose margins are clear of tumour cells) with no expected major sequelae, saw no clear indication for an adjuvant imatinib therapy taking also the size of the tumor into consideration. Gastric tumors have a more favourable prognosis than the intestinal ones, and according to risk stratification based on location, size, and mitotic activity, this GIST carried an intermediate risk for recurrence in the absence of tyrosine kinase inhibitor therapy [7].

Current consensus of the experts at that time was to recommend adjuvant imatinib for patients at high risk of relapse and not for those at low risk of relapse. But there was no consensus for patients at intermediate risk such as our patient [8, 9].

Metastatic GISTs are however associated with poor survival. GISTs most frequently metastasize within the abdominal cavity, especially to the liver and peritoneum, with bone and lung metastases being uncommon sites [1, 10]. Very rare metastasis to the skeletal muscle, adrenal gland [11], brain [12], and testicles [13] is also reported.

Cardiac metastasis after a traditional narrative review using PubMed is reported to our knowledge in only 3 cases so far: 2 cases of metastasis in the left ventricle [6] and one in the right atrium [11] which coincides with the localisation of our case. The initial recognition of the mass was always accidental in CT scan. The differentiation from a thrombus is difficult because intracardiac tumors appear in CT scan as a low-attenuation filling defect, similar to a thrombus [14]. In one report, the differentiation was established through positron emission tomography (PET). PET showed that the mass was metabolically active and a thrombus was by this means excluded. However, PET is certainly not always available and could require patient transportation which may not be possible as the patient may be unstable. In our case, failed improvement after anticoagulation, TEE, and cardiac MRI consolidated the suspected diagnosis of a GIST metastasis which was verified after surgery. In the 3 reported cases of cardiac metastasis so far, the masses diagnosed in the left ventricle and right atrium, respectively, appeared to be attached to the cardiac wall and were therefore consistent with metastatic GIST; however, there is neither histological evidence of GIST nor histological evidence of infiltration of the endocardium or myocardium as the patients underwent no surgical therapy due to very limited prognosis. In our patient, surgery was endorsed because of the high risk for an obstructive form of a cardiogenic shock which could lead to a sudden circulatory deterioration of the patient and because of the possibility for a good response to imatinib therapy. This is the only case reported with histological analysis of a suspected cardiac metastasis of GIST. In our case, because of the lack of endocardial and myocardial infiltration, there is no evidence of hematologous or lymphatic spread of the GIST to the heart in the form of a classic cardiac metastasis and the cardiac involvement of the GIST is due to a contiguous extension of a liver metastasis to the IVC and the right atrium.

Additionally, of clinical interest is the appearance of a new smaller cardiac mass in the right atrium only one week after surgical resection of the tumor. This is the first report of surgical removal of a GIST from the heart, and it shows that early recurrence of cardiac tumors involving GIST after surgery is possible. This experience should be taken into consideration for similar future decisions questioning the meaningfulness of a surgery in advanced cases before implementing KIT-selective tyrosine kinase inhibitor therapy (wait and see how the patient responds to KIT-selective tyrosine kinase inhibitor therapy before cardiac surgery).

The incidence of cardiac metastases is reported in literature, which is highly variable, ranging from 2.3% to 18.3% [15]. This case in addition to the so far published cases of cardiac metastasis involving GIST underlines the importance of considering GIST as a possible cause of cardiac metastasis or cardiac deterioration, associated with a very unfavourable prognosis and a possible high recurrence after surgery. GIST patients should therefore routinely undergo TTE by the time of diagnosis and in follow-up examinations, and cardiac surgery should be considered as the last resort in the battle against metastasized GIST.
